# Knowledge and care seeking practices for ear infections among parents of under five children in Kigali, Rwanda: a cross-sectional study

**DOI:** 10.1186/s12901-017-0040-1

**Published:** 2017-10-10

**Authors:** Kaitesi Batamuliza Mukara, Peter Waiswa, Richard Lilford, Debara Lyn Tucci

**Affiliations:** 1 ENT department, College of medicine and health Sciences, University of Rwanda, and Health Policy, Planning and Management, Makerere University School of Public Health, Kampala, Uganda; 2 Department of Health Policy, Planning and Management, Makerere University School of Public Health, Uganda and Global Health Division, Karolinska Institutet, Stockholm, Sweden; 3 Warwick Medical School, University of Warwick, Coventry, UK; 4 Head and Neck Surgery & Communication Sciences, Duke University, Durham, USA

**Keywords:** Ear infections, Parents, Knowledge, Care seeking, Under five

## Abstract

**Background:**

Infections affecting the middle ear are a common childhood occurrence. Some cases may present with ear discharge through a tympanic membrane perforation which may heal spontaneously. However, up to 5% or more cases of those affected have persistent ear discharge. A number of barriers contribute towards delayed presentation at health facilities for treatment of ear infections. We conducted a study to evaluate parents’ and caregivers’ knowledge and care seeking practices for ear infections in children under five in Gasabo district in Kigali, Rwanda.

**Methods:**

Parents/guardians (*n* = 810) were interviewed using a structured questionnaire to elicit their knowledge of ear infections in children under five and their attitude to seeking care for their children.

**Results:**

The mean age of the respondents was 31.27 years (SD = 7.88, range 17–83). Considering an average of knowledge parameters which included causes, symptoms, prevention, treatment and consequences of ear infections, we found that 76.6% (622) of respondents were knowledgeable about ear infections. We defined a positive practice as seeking medical treatment (community health workers or health facility) and this was found in 89.1% (722) respondents. Correlating knowledge with choice of seeking treatment, respondents were 33% less likely to practice medical pluralism (OR = 0.33, CI 0.11–0.97, *P* = 0.043) if they were familiar with infections. Moreover, urban dweller were 1.7 times more likely to know ear infections compared to rural dwellers (OR = 1.70, CI 1.22–2.38, *P* = 0.002).

**Conclusion:**

The majority of respondents had good knowledge and positive attitudes and practices about ear infection. However, medical pluralism was common. There is need to improve the community’s awareness and access to primary health care facilities for the care of ear infections especially in rural areas of Rwanda.

**Electronic supplementary material:**

The online version of this article (10.1186/s12901-017-0040-1) contains supplementary material, which is available to authorized users.

## Background

Infections affecting the middle ear are a diverse entity and a common childhood occurrence [1–4]. Acute otitis media (AOM) is an inflammation of the middle ear mucosa presenting acutely with symptoms of otalgia and fever [4]. These infections account for the majority of antibiotic prescriptions in young children [3, 4]. The trend of prescribing antibiotics for acute ear infections continues to grow. Guidelines for treatment of AOM have advocated a ‘wait and see’ [3], as up to 80% of those affected show spontaneous resolution [4]. By the 3rd birthday, 80% of children have had at least one episode of acute otitis media [5]. Some cases of acute ear infection may present with ear discharge through a tympanic membrane perforation. The perforation may heal spontaneously in 2–14 days [4]. However, up to 41% of cases have persistent ear discharge; chronic suppurative otitis media (CSOM) [6]. This disease entity also presents with symptoms of tinnitus, hearing loss and persistent discharge refractive to medical treatment usually implying cholesteatoma. Treatment is by antibiotic ear drops and surgery for repair of the persistent tympanic membrane perforation and treatment of complications. Complications are dire and include neck abscesses, mastoiditis, facial nerve paralysis, labyrinthitis, lateral sinus thrombosis, meningitis and brain abscess [4, 7].

It is estimated that 330 million people have CSOM of which 60% have hearing loss; the majority of those affected are children [5]. Hearing loss adversely affects speech and language development and school performance in children [1, 8–10] and may eventually diminish prospects for gainful employment in adulthood. Moreover, 28,000 deaths are attributed to complications due to CSOM [7]. Data on ear infections in sub-Saharan Africa is limited. Published studies estimate CSOM among school going children at 1.6% reported in Tanzania [11] and 2.4% in Kenya [12]. Babigambe (2005) reported a prevalence of 13.2% of CSOM among children aged 6–60 months living in a slum in Uganda [13].

Compelling evidence shows that the painless nature of CSOM renders it an overlooked condition [14–17]. For instance, in their study on prevalence of CSOM among children living in two slums of Dhaka City in Bangladesh, Kamal and Joarder (2014) found that a third of mothers of children examined and found to have CSOM were not aware that their child had this condition, and neither were they aware of the possible sequelae of untreated disease. Moreover, 60% of mothers were not familiar with CSOM nor its treatment and 47% of those who had CSOM, did not seek treatment for it [18].

Risky health seeking practices have been shown to significantly contribute towards an increase in otitis media [19]. In this paper, risky health seeking behaviour is defined as seeking curative services from a source other than that recognised by the Rwandan Health system to treat ear infections. This includes self-medicating with the use of herbs or any other alternative treatment. This definition is modified from the World Health Organisation’s (WHO) working definition [20].

Optimal health seeking behaviour often depends on accessibility of health facilities coupled with knowledge and understanding of the benefit of modern medical treatment as opposed to local customs and beliefs [21–23]. Regarding use of traditional medicine, the WHO states that *“rampant poverty is the prime cause for use, misuse and abuse of traditional medicine”* [20]. Cultural beliefs could supersede education even among people with high literacy levels living in regions with strong cultural beliefs and practices, thus resulting in unhealthy care seeking practices [24].

Unpublished clinical data in Rwanda shows a high prevalence of ear infections in 2013. Up to 21% of patients seen in the ENT department at a referral hospital were diagnosed with ear infections, among whom were young adults who report having infections since childhood. Not only have clinicians noticed delays in presentation for those seeking treatment, but also noted reports of patients using traditional medicine for ear infections regardless of high access to medical insurance. Against this background, we conducted this study to determine the extent of knowledge and care seeking practices of parents and care givers for ear infections in children under 5.

## Methods

### Study design, setting and population

This was a community based study conducted in Gasabo district, one of the 3 districts of Kigali City with a population of 530,907 inhabitants (national census, 2012) and a population density of 1237 persons per square kilometre. It has both rural and urban settlements with 486 villages, 229 of which are rural while 257 are urban [25]. This district is home to one Private referral hospital which offers ENT services among others as well as 3 district hospitals, 17 health centres and 20 functional health posts. According to Integrated Household Living Conditions Survey (EICV3) of 2010/11, the mean transit time by foot to a health facility in Gasabo district is 43.6 min [26]. Each village has 100–150 households and is supervised by 2 Community Health Workers (CHW) called cell coordinators, assisted by 1 assistant cell coordinator.

### Data collection

Using random tables, we sampled 30 villages. CHWs provided a list of households with children aged below 5 years in the sampled villages from which 27 households within each village and an extra 3 households for replacement were sampled. One child from an eligible household was selected using Kish table.

The set exclusion criteria was parents who refused to consent to participate in the study or who refused to give consent for their children to participate. However, no respondent was excluded. A total of 810 parents or guardians were interviewed and 810 children of these parents examined.

We collected data in March 2016. Experienced research assistants carried out data collection while two trained medical students in their ENT clerkship examined the children. We collected data regarding demographics, household characteristics and assets, knowledge, perceptions and care practices of the parents or guardians regarding ear infections. Additional file 1 shows the questionnaire used for data collection.

### Quality control and ethics

Before starting data collection, we held a 1 day training of research assistants where they were familiarised with the study, the questionnaire and data collection procedure. A pre-test session was conducted in a different district of Rwanda and changes incorporated into the pre-designed questionnaire. Our study proposal was submitted and approved by both the College of Medicine and Health Sciences Research Ethics Committee of the University of Rwanda (approval notice: No. 355/CMHS IRB/2015) and the Makerere University School of Public Health Higher Degree Research and Ethics Committee (approval date: 07th December 2015). Parents or guardians with children under 5 in their household provided written consent availed to them both in Kinyarwanda and English to participate in the study.

### Data analysis

We entered data using CSpro 6.2 software after which we performed data cleaning aided by the same software. We used STATA 13.0 for statistical analysis. Given the wide range in the age of respondents, we classified the age into groups with 15 years class interval as follows ≤30, 31–45, 46–60 and >60. Although this last group includes respondents aged 61–83 years, there were only 7 respondents and this was considered a single group.

Knowledge about ear infections and care seeking practices were derived as frequencies and proportions of the responses obtained. Knowledge was subdivided into causes, symptoms, prevention, treatment and consequences of ear infections. The following responses were considered causes of ear infections: poor hygiene, wetting the ears, wax, foreign body and/or microbes. Ear discharge, hearing loss and pain are the cardinal symptoms of ear infections. Respondents who knew 2 of the 3 or any other symptoms were considered knowledgeable of symptoms. Prevention and treatment parameters were rated as ‘yes’ or ‘no’ and respondents who reported ‘yes’ were considered knowledgeable. Respondents who reported hearing loss, persistence of disease, extension into adjacent structures and death as consequences of hearing loss were considered knowledgeable.

We defined a positive practice as seeking medical treatment (CHW or health facility) while positive attitude was derived from responses for choice for treatment seeking and the reason for seeking alternative treatment. Respondents who reported ‘not being worried’, ‘no need for treatment’, and ‘incurable’ were categorised as negative attitude. Moreover choice for information seeking (CHW, health professional) was categorised as positive attitude. To build a knowledge, attitude and practices model, a sum average of each of the responses for each parameter was computed. Additional file 1 gives details of positive and negative ratings for knowledge, attitude and practices.

Socioeconomic status (SES) was derived using Principal Component analysis computed using STATA. We weighted variables of household size and occupancy, source of water supply, types of flooring materials, sanitation facilities and assets. The mean SES was computed and categorised in to Low, Middle and High.

Chi square test and logistic regression analysis were used to derive associations between knowledge of the parents or guardians, SES, residence (urban or rural) and their care seeking practices. The level of significance was set at *p* < 0.05.

## Results

We obtained data for 810 respondents whose results we present. Results from the examination of children will be presented in a subsequent paper.

### Parents’/guardians’ characteristics

The majority of respondents were female; 96% (777). The mean age was 31.27 years (SD = 7.88, range 17–83). A higher proportion of eligible parents took part in the study in urban areas compared to rural areas, accounting for 53% (432) of the study sample. Forty-seven percent (383) of respondents fell in the low SES while 12% (95) were in the high SES category. Fifty eight percent (465) had completed primary school education. More details are shown in Table 1 below.

**Table 1:** Socio-demographic characteristics of the respondents

Characteristic		Frequency (*n* = 810)	Percent
Gender	Males	33	4
	Females	777	96
Age group	≤30	429	52.9
	31–45	346	42.7
	46–60	28	3.5
	>60	7	0.9
Average age	31.27 years		
Residence	Rural	378	46.7
	Urban	432	53.3
Level of education	None	50	6.2
	Primary	465	57.5
	Secondary	220	27.2
	Vocational	74	9.1
Socioeconomic status	Low	383	47.3
	Middle	332	41
	High	95	11.7

### Knowledge and perceptions of ear infections

Sixty-nine percent (561) said they were aware of ear infections, 88.4% (716) said they could be treated while 67.4% (546) said they could actually be prevented. Ear infections were perceived to be caused by poor hygiene in 50.4% (408) of respondents and water entering the ear in 27% (219).

Hearing loss was the most common consequence of ear infections. This was reported by 94% (764) of respondents. Other consequences included poor performance in school reported by 26% (212), persistence of the disease, 14% (115), extension of disease to other organs, 13% (106) and death 7% (70). In contrast, 3% (25) respondents said that ear infections have no consequences.

Table 2 above gives details on knowledge among respondents. We computed the average knowledge of respondents considering all knowledge parameters and found that 76.6% (622) of respondents were knowledgeable about ear infections. Moreover, 47% (381) and 8.5% (69) respondents knew two symptoms or three or more symptoms of ear infections respectively.

**Table 2:** Knowledge of respondents on ear infections

		Frequency	Percent
Knowledge of symptoms of ear infections	None	61	7.5
	1 symptom	299	36.9
	2 symptoms	381	47.0
	≥3 symptoms	69	8.5
Knowledge about prevention	Yes	546	67.4
	No	264	32.6
Knowledge about treatment	Yes	716	88.4
	No	94	11.6
Knowledge of causes of ear infections	Yes	525	64.8
	No	285	35.2
Knowledge about consequences of infections	Yes	789	97.4
	No	21	2.6

Using bivariate and multivariate analysis, there was no statistically significant difference in knowledge when we compared age groups, level of education or socioeconomic status. However, rural dwellers were 1.7 times more likely to have knowledge about ear infections compared to urban dwellers both on bivariate and multivariate analysis as shown in Table 3 below. While respondents were a third of times less likely to practice medical pluralism if they knew about ear infections on bivariate analysis, this was not significant at multivariate analysis.

**Table 3:** Knowledge of ear infections

	Knowledge of ear infections			
	Crude OR (CI)	*P*-value	Adjusted OR (CI)	*P*- value
Age group				
≤ 30	1		1	
31–45	0.8 (0.57–1.12)	0.192	0.89(0.62–1.26)	0.511
46–60	2(0.91–4.40)	0.086	2.26(0.10–5.12)	0.064
> 60	0.51(0.61–4.32)	0.54	0.57(0.06–4.92)	0.638
Residence				
Rural	1		1	
Urban	1.7(1.22–2.38)	0.002*	1.69(1.17–2.44)	0.003**
Level of education				
None	1		1	
Primary education	1.48(0.67–3.24)	0.333	1.55(0.68–3.53)	0.228
Secondary & higher	1.92(0.85–4.34)	0.116	1.61(0.67–3.84)	0.24
Vocational education	1.69(0.67–4.25)	0.267	1.31(.48–3.60)	0.483
Socioeconomic status				
Low	1		1	
Middle	1.35(0.95–1.91)	0.095	1.25(0.87–1.80)	0.254
High	1.23(0.72–2.09)	0.446	1.13(0.63–2.01)	0.783
Where they would seek treatment				
None	1		1	
Modern treatment	0.47(0.18–1.24)	0.126	0.45(0.17–1.24)	0.123
Traditional medicine	0.62(0.22–1.77)	0.371	0.71 (0.24–2.10)	0.533
Medical pluralism	0.33(0.11–0.97)	0.043*	0.35(0.11–1.04)	0.06

*significant at bivariate analysis, **significant at multivariate analysis

### Attitudes towards ear infections

A positive attitude was found in 79.6% (645) respondents. This entailed averaged evaluation of the reasons for their reported choice of treatment seeking, their rating of services provided by the health system as well as the source of information.

Respondents in the 31–45 age range were 50% less likely to have negative attitudes compared to other age groups. Similarly, respondents with secondary education or higher and respondents from middle socioeconomic status were less likely to have a negative attitude towards ear infections. Respondents with a positive attitude were 1 in 5 times less likely to seek treatment at both modern and traditional healers compared to those with a negative attitude. These results were significant both on bivariate and multivariate analysis as shown in Table 4 below.

**Table 4:** Attitude towards ear infections

	Attitudes to ear infections			
	OR (CI)	P-value	Adjusted OR (CI)	*P*- value
Age group				
≤ 30	1		1	
31–45	0.59(0.41–0.84)	0.004*	0.54 (0.37–.79)	0.001**
46–60	1.27(0.54–2.96)	0.586	1.19(0.49–2.87)	0.704
> 60	1		1	
Residence				
Rural	1		1	
Urban	0.91(0.65–1.28)	0.6	1.07(0.73–1.61)	0.677
Level of education				
None	1		1	
Primary education	1.02(0.51–2.01)	0.962	0.10(0.49–2.09)	0.965
Secondary & higher	0.44(0.21–0.95)	0.037*	0.41(0.18–0.94)	0.034**
Vocational education	0.67(0.28–1.63)	0.383	0.72(0.27–1.94)	0.516
Socioeconomic status				
Low	1		1	
Middle	0.56(0.38–0.81)	0.002*	0.59(0.39–0.87)	0.009**
High	0.65(0.37–1.16)	0.143	0.80(0.42–1.51)	0.487
Where they would seek treatment				
None	1			
Modern treatment	0.58(0.21–1.58)	0.286	0.39(0.14–1.13)	0.082
Traditional medicine	0.45(0.15–1.38)	0.163	0.12(0.09–0.89)	0.031**
Medical pluralism	0.18(0.05–0.59)	0.005*	0.12(0.04–0.43)	0.001**

*significant at bivariate analysis, **significant at multivariate analysis

### Care seeking practices with regards to ear infections

Practices were established considering where parents sought information and treatment. Positive practices was found in 89.1% (722) of respondents.

While as 96% (780) of respondents would seek treatment from a health facility, 19% (153) would seek help from a traditional healer. The main reason for seeking traditional treatment was poverty (low affordability) 66% (538), ignorance and lack of knowledge in 62% (504) and lack of a health insurance 41% (333). Community health workers and health professionals were the preferred source of health information for 77% (621) and 60% (452) respondents respectively. Table 5 gives further detail.

**Table 5:** Care seeking practices

		Frequency	Percent
Where they seek treatment	Health facility^a^	780	96
	Traditional medicine^a^	153	19
	Community health worker	68	8
	Self-medication	17	2
	I would not seek treatment	8	1
Why they seek alternative therapy	Poverty^a^	538	66
	Ignorance^a^	504	62
	No health insurance^a^	333	41
	Not worried about it	23	3
	No need, its incurable	17	2
	Others^a^	86	11
Source of information	Community Health worker^a^	621	77
	Health professional^a^	452	60
	Family or neighbours	44	5
	Others	28	4
	I don’t need it	8	1
Consequences of infections	Hearing loss^a^	764	94
	Persistence of the disease^a^	115	14
	Extension to other organs^a^	106	13
	Death	70	7
	None	25	3
	Others	16	2

^a^Outcomes with multiple responses

Urban dwellers were almost 2 times more likely than rural dwellers to have health enhancing practices for ear infections (OR = 1.85, CI 1.18–2.90, *P* = 0.008). Comparing education with practices, we found that while respondents with secondary and higher education were twice likely to have positive practices, (OR = 2.08, CI 0.89–4.87, *P* = 0.091), respondents with vocational education were almost 8 times more likely to have positive practices (OR = 7.90, CI 1.62–38.34, *P* = 0.010). Table 6 below elaborates these findings further.

**Table 6:** Practices for ear infections

	Practices of ear infections			
	OR (CI)	*P* value	Adjusted OR (CI)	*P*- value
Age group				
≤ 30	1		1	
31–45	0.74(0.47–1.17)	0.202	0.80(0.42–1.56)	0.519
46–60	0.63(0.21–1.92)	0.42	0.17(0.22–6.25)	0.855
> 60	Not computed			
Residence				
Rural	1		1	
Urban	1.85(1.18–2.90)	0.008*		
Level of education				
None	1		1	
Primary education	1.6(0.74–3.48)	0.232	0.98(0.29–3.25)	0.972
Secondary & higher	2.08(0.89–4.87)	0.091	1.65(0.43–6.41)	0.468
Vocational education	7.9(1.62–38.34)	0.01*	2.57(0.34–19.24)	0.359
Socioeconomic status				
Low	1		1	
Middle	1.48(0.91–2.39)	0.112	1.74(0.87–3.48)	0.121
High	1.4(0.67–2.97)	0.377	1.70(0.50–5.77)	0.394

*significant at bivariate analysis

## Discussion

In this study which assessed knowledge and care seeking practices of parents of under-five children with ear infections, we found that the majority (76.6%) of respondents were knowledgeable about ear infections while 89.1% had positive care seeking practices. We found no relationship between age group of parent and knowledge of ear infections nor care seeking practices. However, parents in the age range of 31–45 years were less likely to have negative attitudes to healthy care seeking compared to other age groups. We found no study looking at the influence of parental age on care seeking practices to compare with our results. Those who practiced medical pluralism or sought traditional treatment attributed it to poverty (low affordability) in 66% while 62% and 41% cited ignorance (lack of knowledge) and lack of a health insurance respectively. These findings are similar to those of Srikanth in their study on knowledge, attitudes and practices with respect to OM in a rural community in India [22], and also Adeyemo [27] in Nigeria where they found low overall knowledge of otitis media. Only 47% of respondents could identify 2 symptoms of ear infections. Ear discharge, hearing loss and pain were the most common symptoms of ear infections reported, while awareness of other symptoms was low. Pain is a key symptom in acute otitis media [2, 28] while ear discharge is an integral pointer to CSOM [2, 29]. This study looked at ear infections globally and the wide range of symptoms is explained by the nature of ear infections. However, responses given depict more knowledge and awareness of CSOM as compared to acute otitis media.

We found a significant difference in knowledge of ear infections among parents living in rural areas compared to urban areas. Parents in rural areas were more likely to seek traditional treatment for ear infections. Our respondents were more likely to seek modern treatment if they were familiar with ear infections, if they had attained secondary education and beyond and if they lived in an urban setting. Consequently, children from rural areas are at a higher risk of delayed presentation or practicing medical pluralism and developing complications. This is alluded to by Shaheen and colleagues who found treatment seeking in favour of traditional medicine [14]. To overcome this, factors found to result in risky health-seeking behaviour in this study like those found in other studies, which include unaffordable health cost, poor point of care service delivery, distance barriers [22, 30], shortage of medicine in health facilities, and lack of involvement of male parents in child care [30] should be addressed. Findings from a study in Nigeria showed that strong traditional beliefs and practices were more important than level of education in making choices on health. Therefore, health promotion activities can go a long way to foster good health seeking practices.

Respondents who sought traditional treatment attributed this to poverty, lack of awareness and lack of insurance. This finding underscores that CSOM is a disease of poverty [31, 32]. Poverty was associated with inability to pay for services as well as failing to meet transport costs even if the child had medical insurance. Currently, adherence to the Community Based Health Insurance is 91% having increased steadily since being rolled out in 2000 [33]. Consequently more people are able to visit health facilities resulting in long queues that may be a discouragement to patients especially farmers. Holding a Community Based Health Insurance did not therefore preclude traditional medicine as an option for care seeking among our respondents. Health communication and education drives are necessary to help parents understand the nature of ear infections and their treatment to enhance care seeking practices.

The varying attitude and willingness to seek medical treatment seen among our respondents might be due to low level of knowledge, poverty, unsatisfactory service provision at point of first contact and delays in referral health system factors such as long queues. This finding is reported in other studies [30, 34]. Markedly, up to 85% of patients with CSOM in a study in Bangladesh preferred to seek over the counter treatment from untrained local village drug vendors instead of seeking help from qualified staff [14]. Overall, the social context may have the upper hand in determining care seeking behaviour. Participatory planning and provision of services involving beneficiaries as well as improvement of services could help increase utilisation of services thus increase awareness, prevention and treatment of ear infections.

The policy and practice implications of these findings include re-thinking inclusion of ear and hearing care among the attributes of CHWs. The WHO has outlined primary ear and hearing care manuals which can be tailored to a local context [35]. The manuals can help to address the lack of awareness and offer guidance to the scope of practice of era and hearing care at the different levels. This will increase awareness among CHWs which will in turn modify care seeking practices among parents. There is need to improve available ear care services at the primary health care facilities to make them the preferred choice of source of care.

### Study limitations

This study has several strengths but also some limitations. It is the first of its kind to be conducted in Rwanda. In addition, it was a large study that included both a rural and urban context meaning that the findings could be representative of Rwanda and similar settings elsewhere. The limitations include the fact that the questionnaire was formulated in English then translated into Kinyarwanda and back. However, Kinyarwanda lack terminologies equivalent to those in English causing some responses to be missed or generalized. For instance, ear discharge is synonymous with CSOM in Kinyarwanda yet that is not the case in English nor in this study. In addition, dichotomising knowledge maybe crude since this could have multiple connotations.

## Conclusions

The majority of respondents had good knowledge and positive attitudes and practices about ear infection. The preferred point for care seeking is heavily dependent upon knowledge of ear infections and less on age of the parent, gender and SES. Parents in rural settings are more likely to practice medical pluralism. While consequences of ear infections are known, parents still cling to risky health seeking practices for their children. More effort should be put in improving health service accessibility and delivery since this is a barrier to health care seeking practices. Medical professionals and CHW should be educated on ear infection since this is the preferred source of information. More research is required to assess the impact of training and health promotion on knowledge of parents and care seeking practices for ear infections.

## Additional files

Additional file 1: Questionnaire designed specifically for this study and used to collect data. Gives details of criteria for ratings of knowledge, attitude and care seeking practices applied in our study. (DOCX 59 kb)

## Abbreviations

AOM: Acute otitis media
CHW: Community Health Worker
CSOM: Chronic suppurative otitis media
ENT: Ear, Nose and Throat department
SES: Socioeconomic status
WHO: World Health Organisation
