# Supplementary material for: Knowledge and care seeking practices for ear infections among parents of under five children in Kigali, Rwanda: a cross-sectional study
Source: BMC Ear Nose Throat Disord. 2017 Oct 10;17:7. doi: 10.1186/s12901-017-0040-1 (PMC5633896; doi:10.1186/s12901-017-0040-1)
Supplement: Supplementary file 1 — Questionnaire designed specifically for this study and used to collect data. Gives details of criteria for ratings of knowledge, attitude and care seeking practices applied in our study. (DOCX 59 kb) [file 12901_2017_40_MOESM1_ESM.docx]

**Appendix 1**

**Questionnaire on Knowledge and practice of parents**

**Knowledge and practice of parents with regards to ear infections among children under five years**

**Identification**

Sector name................................/....../...../ Cell name ............................................/....../....../

Village name ............................./....../...../ Household number .…........................./....../....../ _______________________________________________________________________

1. Age of respondent………………
2. Do you know anything about ear infections? 1.Yes 2. No
3. Does your child have an ear disease

1.Yes 2. No 3. I don’t know

1. What symptoms would a child with ear infections show?

1. Ear discharge

2. Hearing loss

3. Pain

4. None

5.Other ………………………………………………………………..

6. I don’t know

1. What do you believe to be the cause of ear infections?
2. Infection and or Fever
3. Poor hygiene
4. Water in the ear
5. Wax
6. Trauma
7. Foreign Body
8. Poison
9. Spiritual attacks/ witchcraft
10. Contracted in utero or during delivery
11. Others………………………………………………………………
12. I don’t know
13. Who would take care of your child when they have an ear infection
14. Traditional healer/ Medicine
15. Quacks
16. Community Health worker
17. Nurse
18. Medical doctor
19. Self medication
20. I would not seek treatment
21. Others…………………………………………………………………
22. Ear infections can be prevented.
23. I agree
24. I disagree
25. I don’t know
26. Ear infections can be cured
27. I agree
28. I disagree
29. I don’t know
30. What kind of treatment is used to treat the child’s ear?
31. Herbal medicine
32. Prescribed drugs
33. Cleaning the ear
34. Surgery
35. Hearing aids
36. Ear infections can heal without treatment
37. Religious cures
38. Ear infections cannot be cured
39. Others……………………….……………………………………….
40. I don’t know
41. What do you think can be the consequences of ear infections?
42. None
43. Hearing loss
44. Poor school performance
45. Persistence of the disease
46. Extension of disease to other organs including the brain
47. Death
48. Others………………………………….…………………………
49. Why is it that some children are not taken to the health center for treatment?
50. Ignorance
51. No health insurance
52. Poverty
53. Not worried about it/Someone can live with it safely
54. It heals without any treatment
55. No need because none can cure it
56. No reason because it is on and off
57. Others…………………………………………….…
58. How are services offered to you when you visit a health facility?
59. Health providers examine us
60. Health providers don’t examine us
61. We are given medicine that does not treat the illness
62. We are not given timely referrals
63. Others comments……………………………………………………
64. Where do you get health education or information on health matters?
65. Family members or neighbours
66. Media
67. Community Health worker
68. Health professional
69. Church
70. No where
71. Other………………………………………………………………….

**Thanks for your time**

**Appendix II**

**Knowledge, Attitudes and practices rating criteria**

| **Item** | **Positive** | **Negative** |
| --- | --- | --- |
| **Knowledge** |  |  |
| Do you know anything about ear infections? | Yes | No |
| What symptoms would a child with ear infections show? | Ear discharge  Hearing loss  Pain  Other | None |
| What do you believe to be the cause of ear infections? | Infection and or Fever  Poor hygiene  Water in the ear  Wax  Trauma  Foreign Body | Poison  Spiritual attacks/ witchcraft  Contracted in utero or during delivery  Others  I don’t know |
| Ear infections can be prevented | I agree | I disagree  I don’t know |
| Ear infections can be cured | I agree | I disagree  I don’t know |
| What kind of treatment is used to treat the child’s ear? | Prescribed drugs  Surgery  Hearing aids | Herbal medicine Cleaning the ear  Ear infections can heal without treatment  Religious cures  Ear infections cannot be cured  Others  I don’t know |
| What do you think can be the consequences of ear infections? | Hearing loss  Poor school performance  Persistence of the disease  Extension of disease to other organs including the brain  Death  Others | None |
| **Practices** |  |  |
| Who would take care of your child when they have an ear infection | Community Health worker  Nurse  Medical doctor | Traditional healer/ Medicine Quacks  Self-medication  I would not seek treatment  Others |
| **Attitude** |  |  |
| Why is it that some children are not taken to the health centre for treatment? | Poverty | Ignorance  Not worried about it  It heals without any treatment  No need because none can cure it  No reason because it is on and off  Others |
| Where do you get health education or information on health matters? | Media  Community Health worker  Health professional | Family members or neighbours  Church  No where  Other |
